# The Types and Effectiveness of Mobile Health Applications Used in Improving Oral Cancer Knowledge: A Mixed Methods Systematic Review

**DOI:** 10.1002/hsr2.70171

**Published:** 2024-10-28

**Authors:** Kehinde Kazeem Kanmodi, Afeez Abolarinwa Salami, Kamini Shah, Fatemeh Vida Zohoori, Lawrence Achilles Nnyanzi

**Affiliations:** ^1^ School of Health and Life Sciences Teesside University Middlesbrough UK; ^2^ Faculty of Dentistry University of Puthisastra Phnom Penh Cambodia; ^3^ School of Dentistry University of Rwanda Kigali Rwanda; ^4^ Campaign for Head and Neck Cancer Education (CHANCE) Programme Cephas Health Research Initiative Inc. Ibadan Nigeria; ^5^ Department of Oral and Maxillofacial Surgery University College Hospital Ibadan Nigeria; ^6^ Department of Public Health Dentistry Manipal Academy of Higher Education Manipal India

**Keywords:** app, knowledge, mobile health, oral cancer, systematic review

## Abstract

**Background and Aims:**

The global burden of oral cancer (OC) is enormous. Mobile health applications have been found to play a promising role in cancer prevention; however, no known systematic review evidence exists on whether the use of mobile health applications is effective in increasing public knowledge of OC or not. Therefore, this systematic review aimed to synthesize evidence on the types and effectiveness of mobile health applications used for improving OC knowledge.

**Methods:**

This study adopted a mixed methods systematic review design. The review methodology was informed by Joanna Brigg's Institute's PRISMA checklist and the AMSTAR‐2 guidelines. The literature used for this review were obtained through the search of multiple sources, including 12 electronic databases, web sources, and manual searching of the reference lists and citations of the included articles. Quality appraisal of the included articles was done using the Mixed Methods Appraisal Tool, after which relevant data were collected, synthesized, and configured.

**Results:**

A total of three high‐quality articles, from two studies conducted in India, were included in this review. The studies investigated 574 participants, who are predominantly doctors and community members, on two Android‐based mobile health applications (M‐OncoED and Prayaas). Only Prayaas was found to significantly increase OC knowledge among its users (*p* < 0.05). Only M‐OncoED was found to significantly increase the practice of OC screening advice provision among a selected group of users. No other significant finding was reported on the effect of OC knowledge obtained from the use of these applications on clinical, behavioral, and epidemiological outcomes.

**Conclusion:**

Mobile health application‐based education is a highly underutilised but very promising strategy that can be used to improve public knowledge of OC. This strategy needs to be adopted in public education programmes on OC.

## 1 Background

Oral cancer (OC) is a group of neoplastic lesions affecting the lip, oral cavity, and oropharynx [1, 2]. OC is the most common type of cancer in the head and neck region and the fifteenth deadliest human cancer type known to humans [3]. Within the past three decades (1990–2017), the global incidence rate, mortality rate, and disability‐adjusted life years of OC have doubled [3], making it one of the most notorious human cancers of the current century.

The etiology of OC is multifactorial, and several factors have been implicated in its pathogenesis [4]. These factors include tobacco use, harmful alcohol use, human papillomavirus infection, undernutrition, genetic aberrations, ultraviolet radiation, and chronic occupational exposure to some carcinogenic solvents (such as benzene, diethyl ether, and tetrahydrofuran) [4, 5, 6, 7]. However, its two major etiological factors are tobacco and alcohol use [4, 5].

The clinical features of OC are diverse; however, the common features include oral pain, chronic nonhealing oral ulcers, mobile teeth, oral swelling, and oral bleeding [4]. Pertinently, the manifestations of these clinical features have been associated with serious discomforts and poor quality of life among OC patients, making OC a severely debilitating disease [8].

The treatment of OC is multimodal involving surgery, chemotherapy, and radiotherapy [9]. However, the modality of OC treatment depends on the stage of OC clinical presentation, and the stage of clinical presentation of the disease is strongly associated with its prognosis [7]. This implies that late‐stage OC presentation requires a more intensive and costly approach compared to the early‐stage presentation [10]. Due to late presentation, the prognosis of OC is generally poor, with an average 5‐year survival rate below 50% [5, 11]; hence, this is a high‐priority problematic global health situation which requires urgent attention.

Several modifiable factors including illiteracy, superstitious beliefs, poor access to healthcare facilities, medical pluralism, and poverty, have been reported to be responsible for the late clinical presentation of OC patients [12, 13, 14, 15]. Therefore, the need to address the scourge of OC incidence and its late‐stage clinical presentation across various populations through public health interventions targeting all levels of OC prevention cannot be overemphasized [4, 7].

Digital interventions play a significant role in the prevention of cancer, including OC [16, 17, 18]. For example, digital interventions have been used in the education of diverse population groups on cancer risk factors, cancer self‐examination, cancer screening, and cancer surveillance [18, 19]. Digital interventions for cancer education have been delivered using diverse digital media, and the most common media are websites, mobile phones, tablets, and computers [20, 21, 22, 23, 24]. Of all these media, mobile phone‐based interventions, such as the use of mobile applications and short messaging services, were found to be one of the most promising digital interventions because they are effective, relatively cheaper, and more accessible to the public [21, 25].

Notably, several reviews, including systematic reviews, have been conducted to evaluate the applications of diverse forms (including digital and non‐digital) of interventions in cancer education [20, 21, 22, 23, 24]. However, there is a dearth of review evidence on OC education interventions—after a scoping search of PubMed, SCOPUS, PROSPERO, Cochrane Database of Systematic Reviews, and the first 100 hits on the Google Scholar database, only one systematic review, by Ahuja et al. [26], was found to have evaluated such evidence.

Furthermore, Ahuja et al.'s review only evaluated the effectiveness of interventions on OC knowledge. Their review evaluated randomized control trials and nonrandomised/quasi‐experimental studies which were published between 1989 and 2019. Second, none of the studies included in their review was on a mobile application‐based intervention. Third, their review did not include qualitative studies. The exclusion of observational studies (such as surveys, qualitative studies, before and after studies, case‐control studies, and cohort studies), gray literature, and qualitative studies is an important limitation of the review by Ahuja et al. [26, 27, 28, 29]. Although the findings reported in gray literature are less reliable than journal literature because they are often not peer‐reviewed before their publication, their inclusion into a systematic review tends to increase the comprehensiveness of such a review and facilitate a robust and balanced picture of the available evidence [28]. The inclusion of peer‐reviewed observational studies in a systematic review could provide empirical findings that are similar to experimental studies [27, 29]. Also, the combined use of qualitative and quantitative primary evidence in a systematic review tends to provide more comprehensive evidence needed for the in‐depth understanding of a phenomenon [30, 31, 32].

Based on the observed limitations of the only identified existing review evidence on OC knowledge [26], there is a need to conduct a focused and more comprehensive systematic review to synthesize both empirical and gray evidence, which will include more recent evidence (i.e., those literature that were published in 2020 to date), to synthesize existing qualitative and quantitative evidence, and identify existing knowledge gaps, concerning the types and effectiveness of mobile health applications used in improving OC knowledge [33]. The need for such mixed methods systematic review will provide deeper and broader insights concerning the use of mobile health application‐based strategies in OC education [30, 31, 32]. Ultimately, the outcomes of this review will help to shape the development of research, and evidence‐based practice and policy in this area of research interest [34].

Therefore, this mixed methods systematic review aimed to review the types and effectiveness of mobile health applications used for improving OC knowledge. The primary objectives of this review are to review the types of existing mobile health applications used for improving OC knowledge and to review the effects of the use of such applications on OC knowledge improvement. However, the review's secondary objectives are to review the effects of the use of such applications on clinical, behavioral, and epidemiological outcomes, as knowledge has been reported in the literature to improve these outcomes of interest concerning human cancers [35, 36].

## 2 Materials and Methods

### 2.1 Title and Protocol Registration

The title and protocol of this mixed methods systematic review have been registered with the International Prospective Register of Systematic Reviews (PROSPERO) [CRD42023404119].

### 2.2 Review Design

This review was conducted based on the Joanna Brigg Institute's prescribed recommendations for conducting mixed methods systematic review, and the documentation of the review process was in accordance with the Preferred Reporting Items for Systematic Reviews and Meta‐Analyses (PRISMA) checklist [30, 32, 37, 38]. Also, to accomplish a high‐quality review process, the AMSTAR‐2 (A Measurement Tool to Assess Systematic Reviews, version 2) guidelines informed the methodological conduct of this review [39].

### 2.3 Review Question

This mixed methods systematic sought to address these two research questions:

a. What types of mobile health applications are used for improving OC knowledge?
b. How effective are the available mobile health applications used for improving OC knowledge?

### 2.4 Eligibility

The inclusion and exclusion criteria used in this review were developed using the PICOS (P – Population, I – Intervention, C – Comparison, O – Outcome, S – Study design) framework [40]. Below are these criteria (Table 1):

**Table 1:** The inclusion and exclusion criteria of the review.

PICOS framework	Inclusion criteria	Exclusion criteria
Population	Any population in the world, regardless of race, gender, age, belief, or any other sociodemographic characteristics	Animals
Intervention	Education on oral cancer delivered through an mHealth application	a. Education on nonoral cancers (e.g., breast cancer, prostate cancer, lung cancer, etc.) delivered through an mHealth application. b. Education on oral cancer delivered through means other than non‐mHealth application (e.g., website, health talk, pamphlets, posters, etc.).
Comparison	Control group(s): any group. Comparator group(s): group(s) that received mHealth‐based educational intervention on oral cancer.	Both control group(s) and comparator group(s) did not receive mHealth‐based educational intervention on oral cancer.
Outcome	Knowledge outcomes, behavioral outcomes, and epidemiological outcomes	Other forms of outcomes
Study design	All forms of empirical research designs (randomized quantitative studies, nonrandomized quantitative studies, qualitative studies, and mixed methods studies)	All forms of nonempirical research designs (systematic reviews, scoping reviews, bibliometric reviews, letters to the editor, commentaries, opinions, etc.)

### 2.5 Literature Search Strategy

The literature search was based on the PEO (P – Population; E – Exposure; and O – Outcome) framework (Table 2). In this study, the population of interest was individuals of all sociodemographic characteristics, the exposure of interest was the use of mobile applications on OC knowledge, and the outcomes of interest were knowledge, clinical, behavioral, and epidemiological outcomes. With the support of a Teesside University librarian, the search terms used for this review were obtained from the Medical Subject Heading (MeSH) dictionary and Thesaurus. The literature search involved multiple sources to ensure a comprehensive search was accomplished, and it was done with the aid of the identified search terms, Boolean operators (“AND” and “OR”) and truncations to retrieve all relevant research literature on the use of mobile health interventions in OC knowledge:

**Table 2:** Database search strategy.

		Search query			
PEO framework	Search objectives	Tag	Search Field	Search Terms with truncations	Boolean operator
Population	To search for all population groups. Since all population groups will be included, there is no need for a search on this as such may restrict the search yields	1			
Exposure	To search for papers where participants were exposed to mobile health applications used for increasing knowledge of oral cancer	2	Title and abstract	Oral cancer, Oral squamous cell carcinoma, Oropharyngeal cancer, Oral cavity cancer, Mouth cancer, Lip cancer, Cancer of the lip, Oral malignant neoplasms*, OSCC	OR
		3	Title and abstract	Digital, GSM, Text messages, SMS, MMS, Mobile phone, Mobile health, mHealth, Cell phone, Telecommunications, Apps, Applications, Programmes, Android, iPhone, Smart phone, Technology, Tablet, Tabloid, iPad, Device, Technology enhanced	OR
Outcome	To search for studies that investigated knowledge and awareness	4	Title and abstract	Know*, Aware*, Educat*, Englighten*, Behav*, Teach*, Learn*, Lectur*, Promot*	OR
All	To search for studies that investigated the effectiveness of mobile health applications in improving awareness and knowledge of oral cancer	5		Tag 1 Tag 2 Tag 3 Tag 4	AND

#### 2.5.1 Electronic Research Databases

Without limiters, 10 electronic research databases, including PubMed, SCOPUS, Dentistry and Oral Sciences Source, AMED – The Allied and Complementary Medicine Database, Child Development & Adolescent Studies, SPORTDiscus with Full Text, APA PsycArticles, Psychology and Behavioral Sciences Collection, APA PsycInfo, and CINAHL Ultimate, were searched. (Tables S1–S3).

#### 2.5.2 Databases of Gray Literature and Web Sources

Two databases of gray literature (Google Scholar and MEDNAR) and the webpages of key bilateral and multilateral health organizations (World Health Organization [WHO], West Africa Health Organization [WAHO], United States Agency for International Development [USAID], United Kingdom's National Health Service [NHS], Oral Cancer Foundation [OCF], Centers for Disease Control and Prevention [CDC], and World Dental Federation [FDI]) were also searched to retrieve any relevant literature (Table S4).

#### 2.5.3 Reference Lists and Citations

The reference lists, as well as the citations (obtained from Google Scholar) of eligible literature obtained from the above sources, were manually searched to identify any other eligible literature that was not captured from these sources. Notably, this search was done after literature obtained from other sources had been screened and eligible literature had been identified.

### 2.6 Deduplication of Literature

All literature retrieved from the electronic research database search was deduplicated, and the Rayyan web application was used for the procedure [41].

### 2.7 Literature Screening and Selection

All the deduplicated literature retrieved from the electronic research databases was screened with the aid of the Rayyan web application [41], while those obtained from other sources were screened manually. The screening process was two‐staged, based on a set of inclusion and exclusion criteria, and conducted by two independent reviewers (K.K.K. and A.A.S.). The first stage involved title and abstract screening to determine the relevance of the deduplicated literature at prima facie; at this stage, those literature that were considered nonrelevant were excluded while the residual literature was subjected to a second‐stage screening. In the second stage, full‐text literature evaluations were done to determine their actual relevance for inclusion in the review. Only the eligible literature was finally included in the review.

### 2.8 Quality Appraisal (Risk of Bias Assessment)

All the included original research articles were assessed for risk of bias (quality) using the Mixed Methods Appraisal Tool (MMAT) version 2018 [42]. The AAOCDS (Authority‐Accuracy‐Objectivity‐Coverage‐Date‐Significance) tool, an appraisal tool for gray literature [43], was intended to be used for risk of bias assessment in this review; however, it was not used because no relevant gray literature was included in the review.

The MMAT version 2018 assesses the risk of bias in an original research article using a set of 7‐item questions, of which two questions were general questions for all empirical research designs while five questions were empirical research design‐specific, assessing qualitative, quantitative randomized control trial, quantitative nonrandomised, quantitative descriptive and mixed methods design differently [42].

Using the set of 7‐item questions in the MMAT version 2018, the assessment outcome was graded on a scale of 0–7, with those with a score of 4 and above were considered to be of sound methodological quality. For each appraisal question in the MMAT version 2018, there are three possible answers which can be scored: “yes,” “I can't tell,” and “no” [42]. In this review, a “yes” answer was scored 1, a “I can't tell” or “no” answer was scored 0. The cumulative score per assessed article was summed up and graded on a scale of 0–7.

### 2.9 Data Extraction

Data, including the author's names, year of publication, country/region of study, study setting, study design, sample size, participant characteristics, study instruments, type of mobile health application, and relevant study findings and conclusions, were extracted from all the appraised articles using bespoke data extraction sheets for quantitative and qualitative studies.

### 2.10 Data Synthesis and Configuration

The data synthesis approach used in this mixed methods systematic review was a segregated approach; hence, the findings obtained from quantitative studies (as well as the quantitative part of mixed methods studies) and qualitative studies (as well as the qualitative part of mixed methods studies) were synthesized separately [30, 44]. The narrative synthesis approach was used for the synthesis of quantitative findings, while the thematic analysis approach was used for the synthesis of the qualitative findings [45, 46]. In this review, the meta‐analysis approach was intended to be conducted for the synthesis of quantitative findings; however, it could not be conducted because none of the included articles met the eligibility for meta‐analysis due to methodological heterogeneity reasons [46, 47].

After the completion of the data syntheses, the configuration of both quantitative and qualitative syntheses was done to determine if both syntheses confirm, complement, or refute each other [30].

### 2.11 Ethical Considerations

This study was conducted in accordance with the 1964 Helsinki Declaration on health research involving human or animal subjects. Being a systematic review, this study did not involve data collection from human or animal subjects; hence, prior ethical clearance to conduct this study is not applicable.

## 3 Results

### 3.1 Literature Search and Screening Outcomes

**Figure 1:**
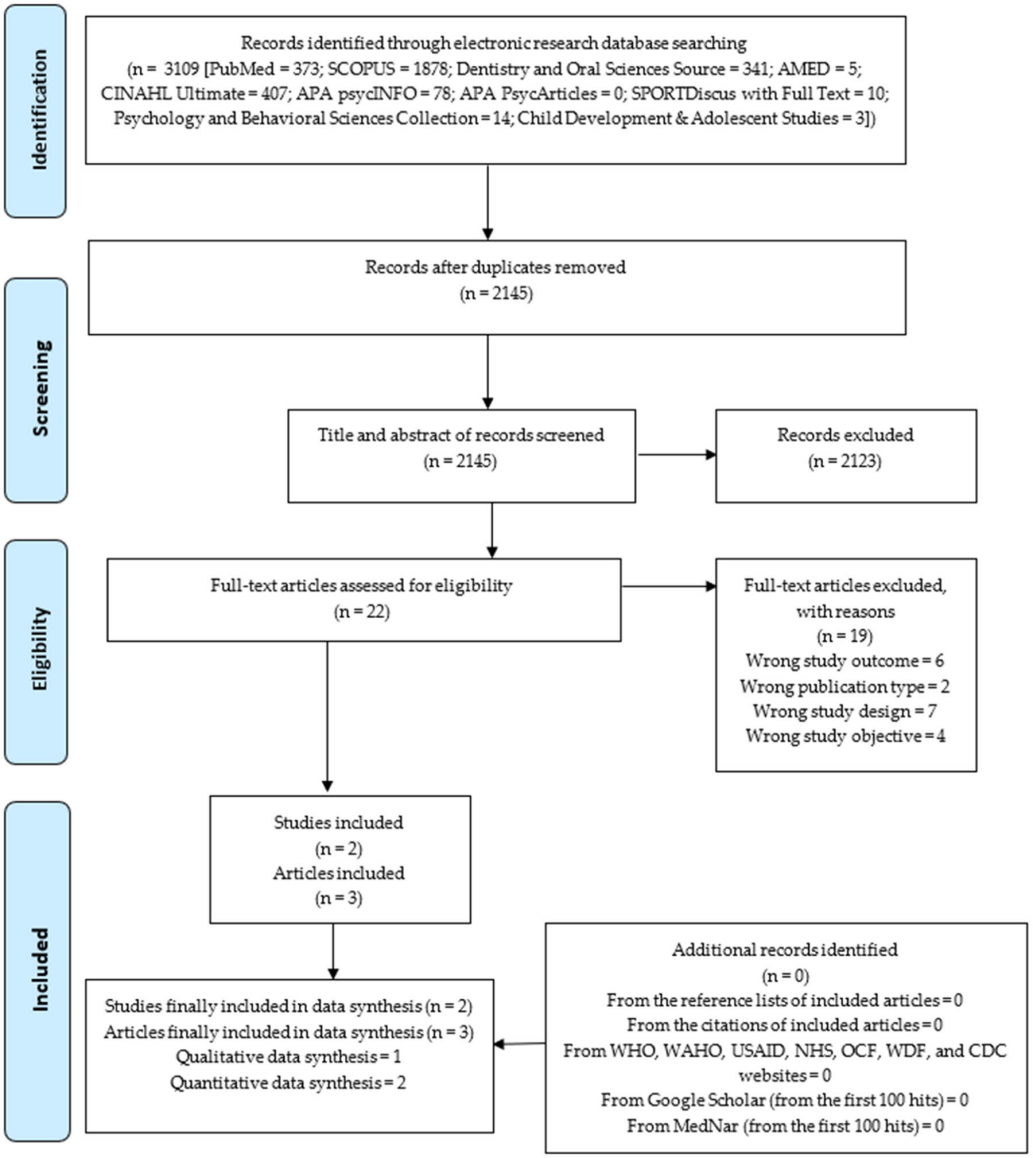
PRISMA flow chart.

A total of 3109 literature were retrieved from the searched electronic research databases. Nine hundred and sixty‐four literature were identified as duplicates and were deleted. From the title and abstract screening of the remaining 2145 deduplicated literature, 2123 nonrelevant literature were excluded, leaving 22 literature (Table S5) for full‐text screening. After the full‐text screening, only three articles from two studies were included (Figure 1).

Additionally, from the manual searches of the websites of notable organizations, the first 100 records retrieved from Google Scholar and MedNar database search, and the reference lists and citations of the 3 included articles, no other eligible article was identified for inclusion into this review.

Finally, a total of three articles from two studies were included in the review (Figure 1).

### 3.2 Publication Trend by Year

All the included studies were published within the past 4 years (2019–2022), with each published per year except for the year 2020 (Table 3).

**Table 3:** Outcomes of risk of bias assessment of the included articles.

			MMAT version 2018 questions									
			Screening questions (S)		Questions specific to study design							
No.	References	Study design	S1	S2	1st	2nd	3rd	4th	5th	Total score	Grading (over 7)	Status
1.	Jose et al. [48]	Qualitative	Yes	Yes	Yes	Yes	Yes	Yes	Yes	7	7/7	MS
2	Subramanian et al. [49]	Quantitative nonrandomised	Yes	Yes	Yes	Yes	Yes	No	Yes	6	6/7	MS
3	Deshpande et al. [50]	Quantitative nonrandomised	Yes	Yes	No	Yes	Yes	No	Yes	4	4/7	MS

*Note:* Yes – 1.0 point; I can't tell (ICT) – 0 point; No – 0 point; MS – Methodologically sound.

### 3.3 Study Designs

Of the three included articles, only one article [48] adopted a qualitative design, while the remaining two articles [49, 50] adopted a quantitative nonrandomised design (Table 3).

### 3.4 Quality Appraisal Outcomes

Also, after the risk of bias assessment, all the included articles were found to be of methodological sound quality (Table 3).

### 3.5 Country of Study

All the included articles were on studies conducted in India (Table 4).

**Table 4:** Summary of the included articles.

No.	References	Country of study	Study setting	Study design	Sample size	Participants' characteristics	Study instruments	Name (and type) of mHealth app	Relevant conclusions
1	[49]	India	Hospital	Quantitative nonrandomised	351 (Broad‐scale recruitment group [physicians of any specialization] = 316; targeted recruitment group [primary care physicians] = 35)	Physicians; 76.9% (270/351) were below the age of 40 years; 41.6% (146/351) were females; 64.4% (226/351) worked in government facility	App	Oncology Education and Training for Providers using Mobile Phones (M‐OncoED) (Android‐based; works online)	The study demonstrated the feasibility and utility of the use of mobile health app in educating physicians on oral cancer.
2	[50]	India	Community (industrial)	Quantitative nonrandomised	50	Industrial employees with mean ± SD age of 41 ± 12 years; mixed genders (37 males and 12 females); all had school education	Prevalidated questionnaire	Prayaas—Oral Cancer Prevention, Treatment, and Rehabilitation (Android‐based, can work online/offline on basic handsets)	Mobile health applications can be used to increase knowledge of rural populations on oral cancer.
3	[48]	India	Hospital	Qualitative	173	Experts in cancer care, doctors, representatives from nongovernmental organizations; and community members. No data on the gender distribution of the participants.	Not categorically stated	Oncology Education and Training for Providers using Mobile Phones (M‐OncoED); (Android‐based; works online)	The study provided evidence on the use of mHealth applications in training healthcare providers to improve oral cancer care.

### 3.6 Study Settings

Two [48, 49] of the included articles reported studies conducted in hospital settings, while the third article [50] was on an industrial community setting (Table 4).

### 3.7 Study Population Characteristics

The population investigated in the included articles were industrial employees [50], doctors [48, 49], other “experts in cancer care” [48], representatives from nongovernmental organizations [48], and community members [48] (Table 4).

A total of 574 participants were investigated in the three articles, of which the majority (61.1%) were investigated by Subramanian et al. [49] (Table 4).

More than 47.0% (270/574) and at least 27.5% (158/574) of the investigated participants were under the age of 40 and females, respectively (Table 4).

### 3.8 Study Instrument

Only two articles [49, 50] categorically reported the study instrument used in investigating their study participants. Of these two articles, only the one by Deshpande et al. [50] categorically stated that a prevalidated questionnaire was used (Table 4).

### 3.9 Synthesized Findings on the Investigated Mobile Health Applications

In total, two mobile health applications were investigated in the three included articles: “Oncology Education and Training for Providers using Mobile Phones (M‐OncoED)” (“M‐OncoED”) [48, 49] and “Prayaas—Oral Cancer Prevention, Treatment, and Rehabilitation” (“Prayaas”) [50] (Table 4). The technical specifications, functionalities, target users, contents, and effectiveness of each of these applications are presented below:

A. M‐OncoED

i. Technical specifications and functionalities
  M‐OncoED is an Android‐based educational application which could work online [48, 49]; however, it was not reported whether the application could also work offline and on basic handsets [48, 49] (Table 4).
ii. Target user
  M‐OncoED was designed specifically for physicians [48, 49] (Table 4).
iii. Contents: Focus on OC
  The educative content of M‐OncoED was not specifically stated in the reviewed articles; however, the application focused on cervical, breast, and oral cancer [48, 49].
iv. Effectiveness

a. Knowledge outcomes
  In the quantitative study by Subramanian et al. [49], the use of M‐OncoED did not yield a significant increase in the OC knowledge scores of its users (*p* > 0.05), although there was a slight increase in such score among those users in the targeted recruitment group (76.96% vs. 80.43%) while there was a decrease in such score among those users in the broad‐scale recruitment group (76.71% vs. 76.2%) (Table 4).
  In the qualitative study by Jose et al. [48], it was reported that the M‐OncoED application provided opportunity for the study participants to have further insights on OC (Table 4).
b. Clinical, behavioral, and epidemiological outcomes
  In the quantitative study by Subramanian et al. [49], it was reported that the usage of M‐OncoED significantly increased the proportion of those of its users, who were categorized under broad‐scale recruitment group, in providing advice on OC screening (73.1% [preuse] vs. 82.9% [postuse], *p* < 0.05); however, its usage did not significantly increase OC screening practices among them (56.3% [preuse] vs. 82.9% [postuse], *p* > 0.05). Among its users, who were categorized under the targeted recruitment group, the application did not statistically significantly increase the practices of providing OC advice (68.6% [preuse] vs. 73.7% [postuse], *p* > 0.05) and OC screening (42.9% [preuse] vs. 47.4% [postuse], *p* > 0.05).
  However, no quantitative data was reported on the effectiveness of M‐OncoED on epidemiological outcomes. Also, no qualitative finding on the effect of the usage of M‐OncoED on clinical, behavioral, and epidemiological outcomes was reported in any of the included articles [48, 49, 50].

B. Prayaas

i. Technical specifications and functionalities
  Prayaas was an Android‐based educational application; it could work online or offline and on basic handsets [50] (Table 4).
ii. Target user
  Prayaas was designed for the lay public, healthcare providers, and patients [50].
iii. Contents: Focus on OC
  Prayaas's educative content was in the forms of texts, pictures, and videos; also, the application focused on OC only [50]. Furthermore, Prayaas contains educative information on tobacco forms, risks associated with tobacco use, tobacco cessation strategies, oral precancer and OC, oral self‐examination, common surgical options for OC, resultant deformities from OC surgery, prosthodontic rehabilitation options, and preradiation consultation [50].
iv. Effectiveness

a. Knowledge outcomes
  No qualitative finding on the effect of the usage of Prayaas on knowledge outcomes was reported in any of the included articles [48, 49, 50]. However, in the quantitative study by Deshpande et al. [50], 98% of the users of Prayaas felt that the application improved their knowledge of tobacco de‐addiction and OC (*p* < 0.0001) while only 80% of them reported that they effectively learnt about self‐oral examination (*p* < 0.0001). These two findings were reported to be statistically significant (Table 4).
b. Clinical, behavioral, and epidemiological outcomes
  No quantitative and qualitative finding was reported on the effect of the usage of Prayaas on clinical, behavioral, and epidemiological outcomes [48, 49, 50] (Tables 4 and 5).

**Table 5:** Thematic analysis of the relevant data extracted from the included qualitative article.

References	Relevant findings		
	First order construct	Second order construct	Third order construct (Theme)
[48]	“This gave an excellent opportunity for me to unlearn many things and learn new things. I never tried to do a clinical breast examination as I was not confident with my skills, instead, I used to advise mammograms for every woman who presented with a lump or was concerned. This module gave an insight into the appropriate early detection methods. I never bothered to examine the oral cavity for any precancerous lesions even if I see a person with stained teeth with the habit of chewing. This training gives me an overview of what I can do even with the facilities at the primary setting.” PCP3_FGD	“Most opined that the app helped them to unlearn and relearn many things. They believed that the knowledge gained was useful for their clinical practice. Majority of participants were thankful that they could receive evidence‐based knowledge directly from the experts. Most of them find app learning an appropriate, cost‐effective, and convenient mode of training.”	Knowledge outcomes

### 3.10 Data Configuration

From the evaluation of both synthesized qualitative and quantitative evidence, it can be concluded that both evidence complement each other.

## 4 Discussion

The findings obtained in this review are noteworthy and are of public health policy implications. This study identified a huge paucity of empirical evidence on the use of mobile health applications in OC education. Only as few as three original research articles from just two studies are available on the systematic review topic area, and all of these articles are from India only. No published study of such has been conducted elsewhere, even in the United Arab Emirates, China, and Libya, which are currently experiencing multiple‐fold increases in their national OC incidence rates [3]. This shows that digital innovations and technologies are highly underutilised in the global public health education strategies on OC. Also, it is not too surprising that India is the country where mobile health applications have been used to improve public awareness and knowledge of OC; this is probably because India is the home to about one‐third of global OC cases and the country is a digital technology hub [51, 52, 53].

Based on this review's findings, the population groups that were investigated using the app were predominantly cancer experts while only a minority were community members and industrial employees. None of the reviewed studies were conducted predominantly or specifically among tobacco smokers, alcohol users, those who engage in oral sex, and areca/betel nut users—all of which are population groups that are highly at risk of OC [5, 54]. It is paramount to test the use of educative mobile health‐based applications on these population groups, as they are potential OC patients [5, 54]. Conducting such studies among them is very crucial as findings from such studies will largely inform the adoption of mobile health‐based applications in both local and global public health strategies against OC.

Based on this review findings, only two educative mobile health applications were found to have been empirically investigated concerning their roles in improving OC knowledge and awareness, of which only one of them was specifically for OC while the other was for OC and other cancer types. Notably, the first application—Prayaas—only focused on tobacco‐associated OC with virtually no focus on OC caused by other major risk factors: areca/betel nut, alcohol, and oral sex (a major cause of oral human papillomavirus infection) [5, 54]. However, based on the very little information reported on the other application—M‐OncoED—it is very difficult to ascertain if the application covered the above‐identified risk factors.

From this review, it was also observed that there is a paucity of evidence concerning the effect of educative mobile health applications on the knowledge, clinical, behavioral, and epidemiological outcomes of OC, especially for qualitative evidence. From the synthesized evidence, it could be identified that educative mobile health applications on OC significantly improved OC knowledge among all investigated populations while they only significantly improved the practice of OC advise provision among selected populations. However, no data was provided on the effect of the use of these applications on OC epidemiology. These identified evidence gaps need to be filled; hence, further studies, especially mixed methods studies, are recommended to investigate these evidence gaps.

However, the reviewed articles have their limitations. First, not all the included articles indicated that a prevalidated or pilot‐tested study instrument was used in the data collection process. Instrument validation is very pivotal in empirical studies, as the use of a validated or pilot‐tested instrument gives more validity and credibility to the findings obtained from such study; hence, the need to interpret the review findings with caution [55, 56]. Second, the information presented about the content of the reviewed mobile health application was inadequate, making it difficult to ascertain, in‐depth, the scope of these applications.

## 5 Limitations of the Systematic Review

This systematic review has its own limitations too. First, the systematic review included only the literature published in English; hence, there is a possibility that some relevant non‐English literature might have been excluded from the review. Second, the review retrieved its literature from only 12 electronic databases (10 research databases, and 2 gray databases), out of several literature databases, and a few other sources. There is a possibility that the print‐only literature, as well as the electronic literature not indexed in the utilized databases, but indexed in other databases, were excluded.

Regardless of the above‐identified limitations, this systematic review has its strengths. First, this review is believed to be the first to systematically review the existing body of empirical evidence concerning the types and effectiveness of mHealth applications in improving knowledge and awareness of OC using a mixed‐methods approach. Second, this study provided in‐depth insights concerning the global evidence landscape on the use of these applications in OC education, which are pivotal to the understanding, development, and use of digital applications in improving OC awareness and knowledge.

## 6 Conclusions and Policy Implications

This systematic review showed that there are only two mobile health educative applications on OC—M‐OncoED and Prayaas—that have been investigated through empirical research, of which one of these applications was designed for physicians. Furthermore, the two applications were highly limited, as they did not seem to provide holistic information on the risk factors and preventive measures of OC. Also, very little to nothing is known about the effect of the use of these applications in improving knowledge, and clinical, behavioral, and epidemiological outcomes on OC. There is a need for further empirical studies to investigate the effects of the use of these applications. Lastly, there is a need for the development of a more comprehensive mobile health application that will provide educative information on the risk factors and preventive and curative measures of OC.

## Author Contributions

**Kehinde Kazeem Kanmodi:** conceptualization, investigation, funding acquisition, writing–original draft, methodology, validation, visualization, writing–review and editing, software, formal analysis, project administration, resources, data curation. **Afeez Abolarinwa Salami:** investigation, methodology, validation, visualization, data curation. **Kamini Shah:** conceptualization, writing–review and editing, supervision, resources, methodology, investigation. **Fatemeh Vida Zohoori:** conceptualization, investigation, methodology, writing–review and editing, supervision, resources. **Lawrence Achilles Nnyanzi:** conceptualization, investigation, methodology, supervision, resources, project administration.

## Ethical Statement

The authors have nothing to report.

## Conflicts of Interest

Kehinde Kazeem Kanmodi is an Editorial Board member of Health Science Reports and a co‐author of this article. To minimize bias, they were excluded from all editorial decision‐making related to the acceptance of this article for publication. The other authors declare no conflicts of interest.

## Supporting information

Supporting information.

## Data Availability

Data sharing is not applicable to this article as no new data were created or analyzed in this study. All authors have read and approved the final version of the manuscript. The manuscript guarantors, Kehinde Kazeem Kanmodi and Lawrence Achilles Nnyanzi, had full access to all of the data in this study and take complete responsibility for the integrity of the data and the accuracy of the data analysis.
