# Supplementary material for: The Types and Effectiveness of Mobile Health Applications Used in Improving Oral Cancer Knowledge: A Mixed Methods Systematic Review
Source: Health Sci Rep. 2024 Oct 28;7(11):e70171. doi: 10.1002/hsr2.70171 (PMC11518889; doi:10.1002/hsr2.70171)
Supplement: Supplementary file 1 — Supporting information. [file HSR2-7-e70171-s001.docx]

**SUPPLEMENTARY FILE**

**Table S1. Search string for PubMed database search**

| **Tag** | **Subject search** | **Search String** |
| --- | --- | --- |
| #1 | Oral cancer | ((((((((oral cancer[Title/Abstract]) OR (oral squamous cell carcinoma[Title/Abstract])) OR (oropharyngeal cancer[Title/Abstract])) OR (oral cavity cancer[Title/Abstract])) OR (mouth cancer[Title/Abstract])) OR (cancer of the lip[Title/Abstract])) OR (oral malignant neoplas*[Title/Abstract])) OR (lip cancer[Title/Abstract])) OR (OSCC[Title/Abstract]) |
| #2 | Mobile health application | (((((((((((((((((((((Digital[Title/Abstract]) OR (GSM[Title/Abstract])) OR (text messages[Title/Abstract])) OR (SMS[Title/Abstract])) OR (MMS[Title/Abstract])) OR (mobile phone[Title/Abstract])) OR (mobile health[Title/Abstract])) OR (mHealth[Title/Abstract])) OR (cell phone[Title/Abstract])) OR (telecommunications[Title/Abstract])) OR (Apps[Title/Abstract])) OR (applications[Title/Abstract])) OR (programmes[Title/Abstract])) OR (android[Title/Abstract])) OR (iPhone[Title/Abstract])) OR (smart phone[Title/Abstract])) OR (technology[Title/Abstract])) OR (tablet[Title/Abstract])) OR (tabloid[Title/Abstract])) OR (iPad[Title/Abstract])) OR (device[Title/Abstract])) OR (technology enhanced[Title/Abstract]) |
| #3 | Education | ((((((((Know*[Title/Abstract]) OR (aware*[Title/Abstract])) OR (educat*[Title/Abstract])) OR (enlighten*[Title/Abstract])) OR (behav*[Title/Abstract])) OR (teach*[Title/Abstract])) OR (learn*[Title/Abstract])) OR (lectur*[Title/Abstract])) OR (promot*[Title/Abstract]) |
| #4 | #1 AND #2 AND #3 | ((#1) AND (#2)) AND (#3) |

**Table S2. Search string for SCOPUS database search**

| **Tag** | **Subject search** | **Search String** |
| --- | --- | --- |
| #1 | Oral cancer | ( TITLE-ABS-KEY ( "oral cancer" )  OR  TITLE-ABS-KEY ( "oral squamous cell carcinoma" )  OR  TITLE-ABS-KEY ( "oropharyngeal cancer" )  OR  TITLE-ABS-KEY ( "oral cavity cancer" )  OR  TITLE-ABS-KEY ( "mouth cancer" )  OR  TITLE-ABS-KEY ( "cancer of the lip" )  OR  TITLE-ABS-KEY ( "oral malignant neoplas*" )  OR  TITLE-ABS-KEY ( "lip cancer" )  OR  TITLE-ABS-KEY ( oscc ) ) |
| #2 | Mobile health application | ( TITLE-ABS-KEY ( digital )  OR  TITLE-ABS-KEY ( gsm )  OR  TITLE-ABS-KEY ( "text messages" )  OR  TITLE-ABS-KEY ( sms )  OR  TITLE-ABS-KEY ( mms )  OR  TITLE-ABS-KEY ( "mobile phone" )  OR  TITLE-ABS-KEY ( "mobile health" )  OR  TITLE-ABS-KEY ( mhealth )  OR  TITLE-ABS-KEY ( "cell phone" )  OR  TITLE-ABS-KEY ( telecommunications )  OR  TITLE-ABS-KEY ( apps )  OR  TITLE-ABS-KEY ( applications )  OR  TITLE-ABS-KEY ( programmes )  OR  TITLE-ABS-KEY ( android )  OR  TITLE-ABS-KEY ( iphone )  OR  TITLE-ABS-KEY ( "smart phone" )  OR  TITLE-ABS-KEY ( technology )  OR  TITLE-ABS-KEY ( tablet )  OR  TITLE-ABS-KEY ( tabloid )  OR  TITLE-ABS-KEY ( ipad )  OR  TITLE-ABS-KEY ( device )  OR  TITLE-ABS-KEY ( "technology enhanced" ) ) |
| #3 | Education | ( TITLE-ABS-KEY ( know* )  OR  TITLE-ABS-KEY ( aware* )  OR  TITLE-ABS-KEY ( educat* )  OR  TITLE-ABS-KEY ( enlighten* )  OR  TITLE-ABS-KEY ( behav* )  OR  TITLE-ABS-KEY ( teach* )  OR  TITLE-ABS-KEY ( learn* )  OR  TITLE-ABS-KEY ( lectur* )  OR  TITLE-ABS-KEY ( promot* ) ) |
| #4 | #1 AND #2 AND #3 | (#1) AND (#2) AND (#3) |

**Table S3. Search string for other database (AMED – The Allied and Complementary Medicine Database; CINAHL Ultimate; Dentistry and Oral Sciences Source; SPORTDiscus with Full Text; APA PsycInfo; APA PsycArticles; Child Development & Adolescent Studies; and Psychology and Behavioral Sciences Collection) search via EBSCO*Host* interface**

| **Tag** | **Subject search** | **Search String** |
| --- | --- | --- |
| S1 | Oral cancer | AB Oral cancer OR AB oral squamous cell carcinoma OR AB oropharyngeal cancer OR AB oral cavity cancer OR AB mouth cancer OR AB cancer of the lip OR AB oral malignant neoplas* OR AB lip cancer OR AB OSCC |
| S2 | Mobile health application | AB Digital OR AB GSM OR AB Text messages OR AB SMS OR AB MMS OR AB Mobile phone OR AB Mobile health OR AB mHealth OR AB Cell phone OR AB Telecommunications OR AB Apps OR AB Applications OR AB Programmes OR AB Android OR AB iPhone OR AB Smart phone OR AB Technology OR AB Tablet OR AB Tabloid OR AB iPad OR AB Device OR AB Technology enhanced |
| S3 | Education | AB Know* OR AB Aware* OR AB Educat* OR AB Englighten* OR AB Behav* OR AB Teach* OR AB Learn* OR AB Lectur* OR AB Promot* |
| S4 | S1 AND S2 AND S3 | (S1) AND (S2) AND (S3) |

**Table S4. Search string for Google Scholar, MedNar, and other website searches**

| **Tag** | **Subject search** | **Search String** |
| --- | --- | --- |
| S1 | Oral cancer | Oral cancer OR oral squamous cell carcinoma OR oropharyngeal cancer OR oral cavity cancer OR mouth cancer OR cancer of the lip OR oral malignant neoplas* OR lip cancer OR OSCC |
| S2 | Mobile health application | Digital OR GSM OR Text messages OR SMS OR MMS OR Mobile phone OR Mobile health OR mHealth OR Cell phone OR Telecommunications OR Apps OR Applications OR Programmes OR Android OR iPhone OR Smart phone OR Technology OR Tablet OR Tabloid OR iPad OR Device OR Technology enhanced |
| S3 | Education | Know* OR Aware* OR Educat* OR Englighten* OR Behav* OR Teach* OR Learn* OR Lectur* OR Promot* |
| S4 | S1 AND S2 AND S3 | (Oral cancer OR oral squamous cell carcinoma OR oropharyngeal cancer OR oral cavity cancer OR mouth cancer OR cancer of the lip OR oral malignant neoplas* OR lip cancer OR OSCC) AND (Digital OR GSM OR Text messages OR SMS OR MMS OR Mobile phone OR Mobile health OR mHealth OR Cell phone OR Telecommunications OR Apps OR Applications OR Programmes OR Android OR iPhone OR Smart phone OR Technology OR Tablet OR Tabloid OR iPad OR Device OR Technology enhanced) AND (Know* OR Aware* OR Educat* OR Englighten* OR Behav* OR Teach* OR Learn* OR Lectur* OR Promot*) |

**Table S5. List of publications whose full texts were screened for inclusion into the review**

| **S/N** | **Citations** | **Decision Status** | |
| --- | --- | --- | --- |
|  |  | Include | Exclude (with reasons) |
| 1 | Song, B., Sunny, S., Li, S., Gurushanth, K., Mendonca, P., Mukhia, N., Patrick, S., Gurudath, S., Raghavan, S., Imchen, T., Leivon, S., Kolur, T., Shetty, V., Bushan, V., Ramesh, R., Lima, N., Pillai, V., Wilder-Smith, P., Sigamani, A., Suresh, A., … Liang, R. (2021). Mobile-based oral cancer classification for point-of-care screening. *Journal of biomedical optics*, *26*(6), 065003. https://doi.org/10.1117/1.JBO.26.6.065003 |  | Yes (Wrong study outcome) |
| 2 | Deshpande, S., Radke, U., Karemore, T., Mohril, R., Rawlani, S., & Ingole, P. (2019). A Novel Mobile App for Oral Cancer Awareness amongst General Population: Development, Implementation, and Evaluation. *The journal of contemporary dental practice*, *20*(2), 190–196. | Yes |  |
| 3 | Jose, R., Subramanian, S., Augustine, P., Rengaswamy, S., Nujum, Z. T., Gopal, B. K., Saroji, V., Samadasi, R., John, S., Narendran, M., Lal, A., & Pillai, R. (2022). Design and Process of Implementation Mobile Application Based Modular Training on Early Detection of Cancers (M-OncoEd) for Primary Care Physicians in India. *Asian Pacific journal of cancer prevention : APJCP*, *23*(3), 937–946. https://doi.org/10.31557/APJCP.2022.23.3.937 | Yes |  |
| 4 | Yadav, K., Hariprasad, R., Gupta, R., Upadhayay, S., Sharma, V., Kumari, M., Mehrotra, R., Kumar, S., Gupta, S., & Singh, S. (2022). Cancer awareness & its association with demographic variables & mobile phone usage among the rural population of a district in north India. *The Indian journal of medical research*, *156*(1), 94–103. https://doi.org/10.4103/ijmr.IJMR_3145_20 |  | Yes (Wrong study outcome) |
| 5 | Ulloa-Morales, Y., Negreira-Martínez, F., Blanco-Hortas, A., Patiño-Castiñeira, B., San-Román-Rodríguez, E., Varela-Centelles, P., & Seoane-Romero, J. M. (2021). Online audio-visual information on oral cancer for Spanish-speaking laypersons. A cross-sectional study. *Medicina oral, patologia oral y cirugia bucal*, *26*(6), e795–e801. https://doi.org/10.4317/medoral.24770 |  | Yes (Wrong study design) |
| 6 | Vaishampayan, S., Malik, A., Pawar, P., Arya, K., & Chaturvedi, P. (2017). Short message service prompted mouth self-examination in oral cancer patients as an alternative to frequent hospital-based surveillance. *South Asian journal of cancer*, *6*(4), 161–164. https://doi.org/10.4103/sajc.sajc_258_16 |  | Yes (Wrong study design) |
| 7 | Haron, N., Zain, R. B., Nabillah, W. M., Saleh, A., Kallarakkal, T. G., Ramanathan, A., Sinon, S. H., Razak, I. A., & Cheong, S. C. (2017). Mobile Phone Imaging in Low Resource Settings for Early Detection of Oral Cancer and Concordance with Clinical Oral Examination. *Telemedicine journal and e-health : the official journal of the American Telemedicine Association*, *23*(3), 192–199. https://doi.org/10.1089/tmj.2016.0128 |  | Yes (Wrong study design) |
| 8 | Wen K. Y. (2020). Not Just Another App: Realizing the Potential of Mobile Healthcare Applications to Promote Adherence to Oral Cancer Therapy Medications. *Journal of the National Comprehensive Cancer Network : JNCCN*, *18*(2), 219–220. https://doi.org/10.6004/jnccn.2020.7532 |  | Yes (Wrong publication type) |
| 9 | Nethan, S. T., John, A., Ravi, P., Dhanasekaran, K., Babu, R., & Hariprasad, R. (2022). Advanced virtual mentoring of dentists in oral cancer screening and tobacco cessation - An interventional study. *Indian journal of dental research : official publication of Indian Society for Dental Research*, *33*(3), 241–246. https://doi.org/10.4103/ijdr.ijdr_8_21 |  | Yes (Wrong study design) |
| 10 | Zain, R. B., Pateel, D. G. S., Ramanathan, A., Kallarakkal, T. G., Wong, G. R., Yang, Y. H., Zaini, Z. M., Ibrahim, N., Kohli, S., & Durward, C. (2022). Effectiveness of "OralDETECT": a Repetitive Test-enhanced, Corrective Feedback Method Competency Assessment Tool for Early Detection of Oral Cancer. *Journal of cancer education : the official journal of the American Association for Cancer Education*, *37*(2), 319–327. https://doi.org/10.1007/s13187-020-01814-1 |  | Yes (Wrong study objective) |
| 11 | Rêgo, T. J. R. D., Lemos, J. V. M., Matos, A. P. L., Caetano, C. F. F., Dantas, T. S., Sousa, F. B., Barros Filho, E. M., & Silva, P. G. B. (2022). Development and professional validation of an App to support Oral Cancer Screening. *Brazilian dental journal*, *33*(6), 44–55. https://doi.org/10.1590/0103-6440202204895 |  | Yes (Wrong study outcome) |
| 12 | Ramesh, R. M., Patrick, S., Lotha, Z., Azole, Aier, A., & Birur N, P. (2022). Prevalence and determinants of oral potentially malignant lesions using mobile health in a rural block, northeast India. *Tropical doctor*, *52*(1), 53–60. https://doi.org/10.1177/00494755211049973 |  | Yes (Wrong study outcome) |
| 13 | Subramanian, S., Jose, R., Lal, A., Augustine, P., Jones, M., Gopal, B. K., Swayamvaran, S. K., Saroji, V., Samadarsi, R., & Sankaranarayanan, R. (2021). Acceptability, Utility, and Cost of a Mobile Health Cancer Screening Education Application for Training Primary Care Physicians in India. *The oncologist*, *26*(12), e2192–e2199. https://doi.org/10.1002/onco.13904 | Yes |  |
| 14 | Graboyes, E. M., Maurer, S., Park, Y., Marsh, C. H., McElligott, J. T., Day, T. A., Hornig, J. D., & Sterba, K. R. (2020). Evaluation of a novel telemedicine-based intervention to manage body image disturbance in head and neck cancer survivors. *Psycho-oncology*, *29*(12), 1988–1994. https://doi.org/10.1002/pon.5399 |  | Yes (Wrong study outcome) |
| 15 | Haron, N., Zain, R. B., Ramanathan, A., Abraham, M. T., Liew, C. S., Ng, K. G., Cheng, L. C., Husin, R. B., Chong, S. M. Y., Thangavalu, L. A., Mat, A., Ismail, H. B., Mahalingam, S. A., & Cheong, S. C. (2020). m-Health for Early Detection of Oral Cancer in Low- and Middle-Income Countries. *Telemedicine journal and e-health : the official journal of the American Telemedicine Association*, *26*(3), 278–285. https://doi.org/10.1089/tmj.2018.0285 |  | Yes (Wrong study objective) |
| 16 | Greer, J. A., Jacobs, J. M., Pensak, N., Nisotel, L. E., Fishbein, J. N., MacDonald, J. J., Ream, M. E., Walsh, E. A., Buzaglo, J., Muzikansky, A., Lennes, I. T., Safren, S. A., Pirl, W. F., & Temel, J. S. (2020). Randomized Trial of a Smartphone Mobile App to Improve Symptoms and Adherence to Oral Therapy for Cancer. *Journal of the National Comprehensive Cancer Network : JNCCN*, *18*(2), 133–141. https://doi.org/10.6004/jnccn.2019.7354 |  | Yes (Wrong study objective) |
| 17 | Birur, N. P., Gurushanth, K., Patrick, S., Sunny, S. P., Raghavan, S. A., Gurudath, S., Hegde, U., Tiwari, V., Jain, V., Imran, M., Rao, P., & Kuriakose, M. A. (2019). Role of community health worker in a mobile health program for early detection of oral cancer. *Indian journal of cancer*, *56*(2), 107–113. https://doi.org/10.4103/ijc.IJC_232_18 |  | Yes (Wrong study design) |
| 18 | Bhatt, S., Isaac, R., Finkel, M., Evans, J., Grant, L., Paul, B., & Weller, D. (2018). Mobile technology and cancer screening: Lessons from rural India. *Journal of global health*, *8*(2), 020421. https://doi.org/10.7189/jogh.08.020421 |  | Yes (Wrong study outcome) |
| 19 | Nayak, P. P., Nayak, S. S., Sathiyabalan, D., Aditya, N. K., & Das, P. (2018). Assessing the Feasibility and Effectiveness of an App in Improving Knowledge on Oral Cancer-an Interventional Study. *Journal of cancer education : the official journal of the American Association for Cancer Education*, *33*(6), 1250–1254. https://doi.org/10.1007/s13187-017-1239-y |  | Yes (Wrong study design) |
| 20 | Lin, T. H., Wang, Y. M., & Huang, C. Y. (2022). Effects of a mobile oral care app on oral mucositis, pain, nutritional status, and quality of life in patients with head and neck cancer: A quasi-experimental study. *International journal of nursing practice*, *28*(4), e13042. https://doi.org/10.1111/ijn.13042 |  | Yes (Wrong study objective) |
| 21 | Lin, H. Y., Chen, S. C., Peng, H. L., & Chen, M. K. (2016). Effects of a case management program on patients with oral precancerous lesions: a randomized controlled trial. *Supportive care in cancer : official journal of the Multinational Association of Supportive Care in Cancer*, *24*(1), 275–284. https://doi.org/10.1007/s00520-015-2787-5 |  | Yes (Wrong study design) |
| 22 | Wu, C. H., Huang, Y. C., Chiang, D. H., Yang, Y. Y., Yang, L. Y., Kao, S. Y., Chen, C. H., & Lee, F. Y. (2020). A quality improvement pilot project of training nurses to use VR educational aids to increase oral cancer patients' pretreatment knowledge and satisfaction. *European journal of oncology nursing : the official journal of European Oncology Nursing Society*, *49*, 101858. https://doi.org/10.1016/j.ejon.2020.101858 |  | Yes (Wrong publication type) |
